# Computational Study of the Structure of a Sepiolite/Thioindigo Mayan Pigment

**DOI:** 10.1155/2012/672562

**Published:** 2012-11-01

**Authors:** Manuel Alvarado, Russell C. Chianelli, Roy M. Arrowood

**Affiliations:** ^1^Materials Research and Technology Institute, The University of Texas at El Paso, El Paso, TX 79912, USA; ^2^Department of Metallurgical & Materials Engineering, The University of Texas at El Paso, El Paso, TX 79912, USA

## Abstract

The interaction of thioindigo and the phyllosilicate clay sepiolite is investigated using density functional theory (DFT) and molecular orbital theory (MO). The best fit to experimental UV/Vis spectra occurs when a single thioindigo molecule attaches via Van der Waals forces to a tetrahedrally coordinated Al^3+^ cation with an additional nearby tetrahedrally coordinated Al^3+^ also present. The thioindigo molecule distorts from its planar structure, a behavior consistent with a color change. Due to the weak interaction between thioindigo and sepiolite we conclude that the thioindigo molecule must be trapped in a channel, an observation consistent with previous experimental studies. Future computational studies will look at the interaction of indigo with sepiolite.

## 1. Introduction

**Figure 1:**
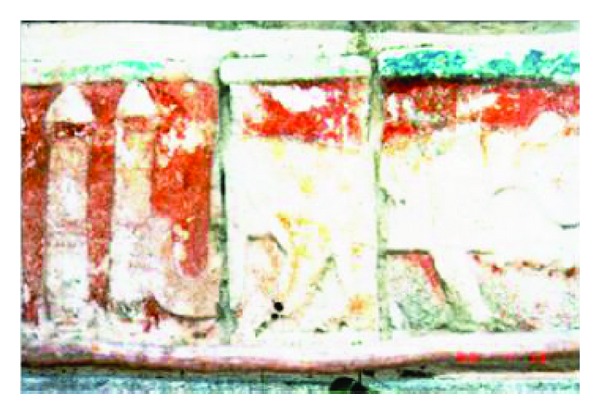
A photograph of a monument in Chichen Itza, YUC (900 C.E.), Mexico, painted with Maya Blue, from [6].

Among the ruins of the Mayan civilization are many examples of murals displaying a very vivid and beautiful blue paint known as Maya Blue (Figure 1). For more than 50 years this pigment has been the subject of much interest and debate among the scientific community [1, 2]. In 1931, Merwin published photographs of the ruins of a mural at Chichen Itza, noting that a blue pigment was very distinct among the other colors present [3]. The term Maya Blue was first coined in 1946 by Gettens and Stout [4] as this pigment was believed to exist exclusively on relics of the Mayan civilization in the Yucatan Peninsula region. Since these first investigations this paint has been identified in many other Mesoamerican artifacts found outside this region but is still known by its original designation, Maya Blue.

This fascinating material, composed of a fibrous clay material (palygorskite) and an organic dye (indigo), has long been renowned for its chemical stability and vivid color. Maya Blue is of great interest due to its resistance to solvents, oxidants, reducing agents, alkalis, extreme humidity, acids, and exposure to ultraviolet radiation. Even more remarkable is the fact that Maya Blue contains no heavy metal content. This material is synthesized using a very simple process involving grinding the clay, mixing with the organic dye, and heating the mixture to a temperature above the boiling point of water (typically 120°C to 190°C), which may have been the method used by Mayan craftsmen [5]. We now know that in addition to indigo a large variety of organic dyes can be used to create similar pigments, with the choice of the dye determining the color of the finished product [6]. Additionally, other phyllosilicate materials (such as sepiolite and montmorillonite) have led to results which are similar as those obtained in palygorskite-based Mayan pigments [7], leading to a situation where an almost innumerable variety of clay/dye mixtures and corresponding colors exist. In order to avoid a brute force approach where every new mixture must be physically synthesized to determine its color there is a strong inspiration to model and predict the color resulting from such new combinations of these materials. This paper will propose a model for the binding for one such material, consisting of the clay sepiolite being combined with the organic dye thioindigo (the choice of material was purely determined by the availability of experimental UV/Vis data in order to validate the computational results).

### 1.1. Sepiolite

**Figure 2:**
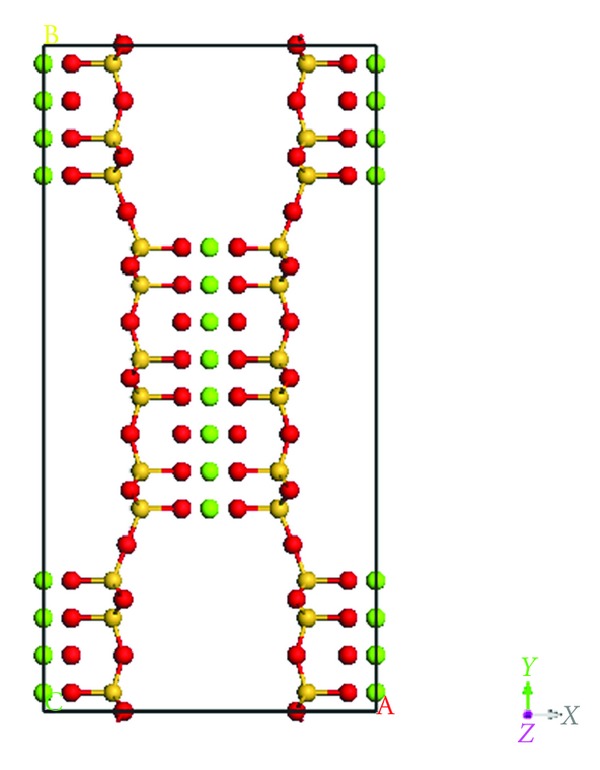
Unit cell of sepiolite, showing magnesium (green) octahedrally coordinated between chains of SiO_4_ (silicon shown in orange, oxygen in white). View is along the *c*-axis.

Sepiolite, like palygorskite, is a fibrous, colorless phyllosilicate material consisting of layers of SiO_4_ tetrahedra (oriented such that unshared oxygen atoms are facing each other) bonded together with octahedrally coordinated magnesium atoms between these tetrahedral sheets [8–10]. The unit cell of the clay, shown in Figure 2, has orthorhombic symmetry with lattice parameters a = 13.5 Å, b = 27.0 Å, and c = 5.30 Å [10]. Like palygorskite, rectangular channels forms parallel the *c*-axis of the unit cell and often contain zeolitic water or hydroxyl groups. A trivalent cation (Mn^3+^, Al^3+^, or Fe^3+^) is often found as a substitutional impurity occupying a silicon site in a tetrahedral sheet [9]. Additional water molecules are present in the octahedral magnesium layers (structural water, as opposed to zeolitic water).

The larger channel size in sepiolite compared to palygorskite makes channel diffusion and adsorption of dye molecules a more probable process in sepiolite. Ovarlez et al. [11] found that indigo did not react with sepiolite until the clay was heated well above the boiling point of water (180°C–550°C), concluding that both zeolitic and structural water groups must be removed from sepiolite before indigo is bonded with sepiolite due to bond energies appearing in FTIR spectra. Giustetto et al. [12] found evidence of chemical bonding between indigo and sepiolite when the clay had been heated to 190°C (enough to remove zeolitic but not structural water) using IR and RAMAN spectroscopy, noting that a weak bond existed between the clay and C=O and N–H functional groups in indigo. Furthermore, this group concluded that this weak chemical bond could show surprising stability when formed within channels where the adsorbed indigo was difficult to reach and thus remove. In these cited studies there was evidence that indigo was present in its dehydroindigo form, allowing more structural flexibility of the dye molecule. As dehydroindigo is a strong chemical analogue to thioindigo we expect thioindigo to diffuse and bond in a similar fashion as dehydroindigo, especially if Van der Waals bonding is the predominant chemical attraction.

### 1.2. Color Changes in Clay/Dye Complexes

**Figure 3:**
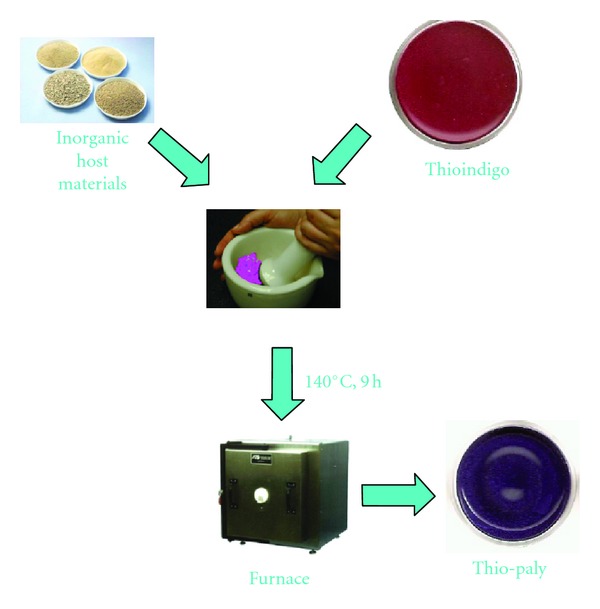
Color change of clay/dye complex during synthesis, in this case palygorskite/thioindigo.

**Figure 4:**
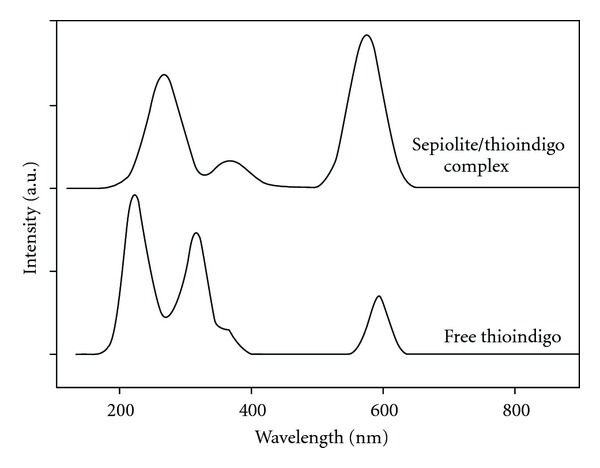
Computational color change in sepiolite-bonded thioindigo. The UV-Vis spectra are displaced vertically for clarity.

A key measure of the formation of the clay/dye complex is a dramatic color change, as shown in Figure 3. These color changes occur only as the temperature of the clay/dye mixture is heated and a chemical interaction takes place. The blue of indigo, for example, changes to the classic Maya Blue color upon heating, a nonreversible change (a strong indication of the presence of the dehydroindigo form of the indigo molecule). Although the figure shows an example of a color change with a palygorskite/thioindigo complex these color changes have been observed for a wide range of both clays (palygorskite, sepiolite and montmorillonite) and dye molecules (indigo, thioindigo, vat orange 5, yellow 33, etc.). This color change is seen as evidence of a chemical interaction between the dye and clay, an assertion confirmed by IR and XRD spectra, HRTEM analysis, and DTA analysis [13]. An example of the color change seen in our computational model is shown in Figure 4.

## 2. Preliminary Chemical Model for a Synthetic Sepiolite/Thioindigo Complex

**Figure 5:**
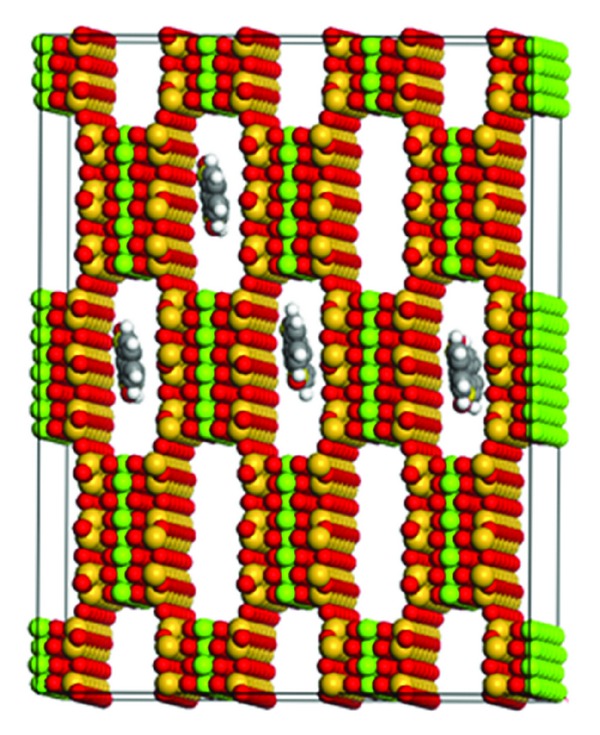
Crystal structure of sepiolite with zeolitic water removed and thioindigo tucked into several channels. The model was generated using the Sorption module in Materials Studio 4.0.

Most studies on the structural characterization of sepiolite-based dye complexes have drawn a parallel with palygorskite-based complexes. For these structures, two significant and related issues are (1) whether surface biding or channel “tucking” is the predominant means of interaction and (2) the presence of zeolitic water in these channels. The process of synthesizing sepiolite-based dye complexes involves mixing the dye with sepiolite and heating the mixture to temperatures above 120°C, resulting in a mass loss of 6–10% in the mixture, which has been attributed to loss of zeolitic water molecules by thermogravimetric analysis (TGA) [14]. Although sepiolite has been observed to undergo structural collapse and loss of channels with removal of structural water [15], the temperatures required to achieve this (approximately 800°C) are far in excess of the temperatures used in the synthesis of this material. We therefore proceed on the assumption that the channels in sepiolite are clear of water and are capable of having thioindigo molecules diffusing into these channels, as the channel size is more than large enough to accommodate the dye molecule. Our preliminary chemical model is shown in Figure 5. 

## 3. Computational Modeling Procedure

The computational methodology used for this study is as follows. The proposed structure was optimized using a plane-wave pseudopotential density functional theory (DFT) code, CASTEP [16]. A Gaussian smearing width of each energy level is introduced to eliminate a discontinuity in the energy when an electron band crosses a Fermi level during calculation. This width is subsequently halved upon the convergence of energy values to the parameter specified by a Self-Consistent Field (SCF) condition, in which the field experienced by an atom depends on the global distribution of atoms. The calculation is then repeated and the process continues in this manner until the smearing width converges to a specific minimum value. The initial smearing width of each calculation was set to 1 eV, as this value offered a reasonable compromise between speed and accuracy. It should be noted that the CASTEP graphic user interface allows for a range of values for the smearing width varying between 4.0 eV and 0.1 eV.

A pseudopotential chosen for these preliminary calculations was a norm-conserving nonlocal pseudopotential as described by Lin et al. [17]. Although additional pseudopotentials are available through the CASTEP GUI, it was decided to limit these initial calculations to a single pseudopotential due to the computation time involved.

The plane-wave expansion of electronic wavefunctions used by CASTEP requires the input of a kinetic cut-off energy for these wavefunctions. It was determined that specifying a cut-off energy of no less than 200 eV provided the optimum balance between computation time and precision, as cut-off energies below this value did not yield significantly varying energies. The resulting constraint on spacing of cells in reciprocal space used to generate k-point by a Monkhorst-Pack scheme 42 was set at 0.07 Å^−1^. These parameters were employed in all calculations.

The CASTEP calculations consisted of both energy calculations for fixed structures and geometry optimizations for structures without fixed parameters. The geometry optimizations in CASTEP involve the movement of atoms in a crystal structure until a geometry is achieved that minimizes the energy. An additional investigation involved the varying of the lattice parameters resulting from each geometry optimization to within 0.1 Å of the optimized values to ensure the convergence of the energy to an absolute as opposed to a local minimum. This procedure was employed in all cases to test the validity of optimized lattice parameters.

For MO calculations, the molecular orbital (MO) method VAMP [18] was used on the nonperiodic version of the organic/inorganic complex. First, the energy of the optimized structure (as optimized in a periodic version of the structure by CASTEP) was calculated. A ZINDO (Zerner's Intermediate Neglect of Differential Overlap) Hamiltonian, which uses INDO to handle differential overlap, was used [19]. A full Configuration Interaction (CI) scheme, which allows all available permutations of electronic excitations in each orbital, was applied. The SCF tolerance in the calculation was 5 × 10^−7 ^eV/atom (fine criterion within the VAMP GUI). Once the energy was calculated for the structure these same constraints were applied to calculate the UV/Vis spectrum using a Gaussian integration scheme and applying a smearing width of 30 nm FWHM in order to simulate instrumental broadening.

## 4. DFT and MO Results for Calculation of Optical Spectra for Sepiolite/Thioindigo Complex

**Figure 6:**
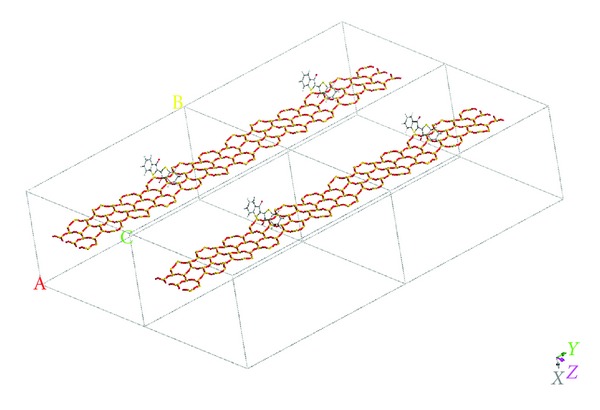
Periodic structure of sepiolite/thioindigo complex used for CASTEP calculation.

We modeled the sepiolite-thioindigo system by first optimizing the structure geometry using CASTEP, and then calculating the optical spectrum using VAMP. The proposed structures (with approximate geometries provided by the Sorption module in Cerius^2^ [20] as a starting point) were modeled as a surface by taking a 14 × 3 lattice of adjacent silica rings (mimicking the morphology along a wall of a channel in sepiolite) and introducing a substitutional impurity at a silicon site (based on spectroscopic data, aluminum, iron, and magnesium were used in our simulations). Using this proposed surface complex as a basis we constructed a three-dimensional unit cell as required by CASTEP for geometry optimizations (Figure 6). By allowing the unit cell to have a very large lattice parameter perpendicular to the surface we are able to simulate a surface interaction while satisfying three-dimensional periodicity required by CASTEP as the simulation becomes one of modeling a series of noninteracting parallel planes (confirmed by the fact that the total electronic energy converged to a constant value as this lattice parameter is increased). Once the model converged to an optimum geometry by CASTEP using a generalized gradient approximation (GGA) functional the structure is reduced to a nonperiodic structure (required for a VAMP calculation) consisting of 3 adjacent silica rings and the attached dye molecule as structures larger than this were prohibitively expensive in terms of computational power. A VAMP molecular orbital simulation employing a neglect of diatomic differential overlap (NDDO) Hamiltonian with a full configuration interaction (CI) scheme was then used to calculate UV/Vis spectra and compare to experimental data. This process was repeated using single and multiple substitutional metallic impurity sites using the aforementioned metals found in sepiolite as well as monomer, dimer, and trimer thioindigo complexes. We found the best agreement between computational and experimental spectra when the structures had the following characteristics:

- tetrahedral bonding of thioindigo to an aluminum impurity site;
- a single thioindigo molecule bonded to an aluminum site in the nonperiodic surface mesh;
- distortion of the thioindigo molecule from its planar structure. This has been associated with the observed color changes in the synthesis of mayan pigments and related materials [13].

**Figure 7:**
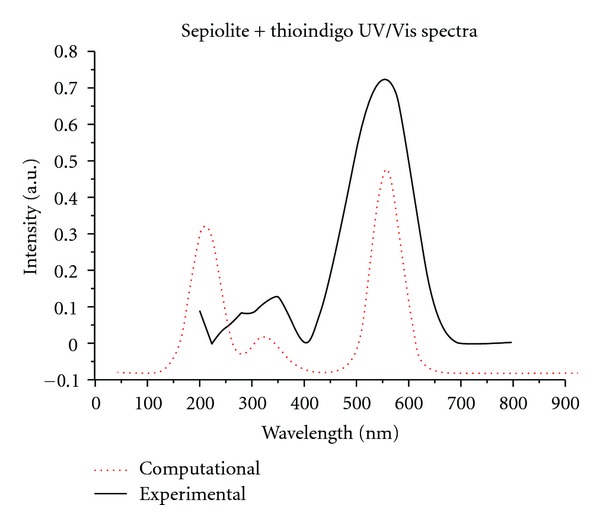
Comparison of computational and experimental UV/Vis spectrum of the thioindigo/sepiolite complex.

**Figure 8:**
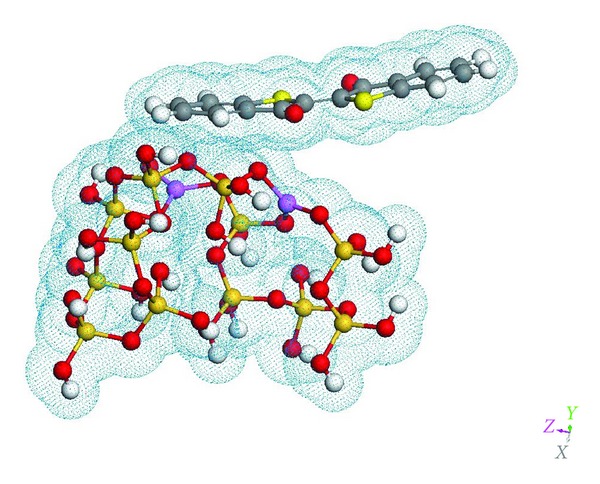
Optimized thioindigo/sepiolite structure with Van der Waals surface displayed. Note the distortion of the planar geometry of the thioindigo molecule.

The first two observations ran contrary to our findings with a palygorskite/thioindigo complex, which saw the best UV/Vis spectral fit for octahedral binding and a dimer thioindigo structure attaching to the surface mesh. The results for the optical spectrum for the thioindigo/sepiolite complex are shown in Figure 7, showing excellent agreement in both visible and near-UV regions between the computational and experimental results. The optimized molecular structure corresponding to this result is shown in Figure 8. Two interesting features of this structure are (1) unlike palygorskite-based mayan pigments which display a dimer structure on the adsorbed dye molecules, only a single dye molecule bonds to the surface mesh, and (2) the dye molecule is bonded to the surface via a Van der Waals interaction. While such an interaction would seem to be at odds with the resistance to color fading of mayan pigments we must consider the added stability of channel bonding sites within the clay as due to physical confinement of the dye molecules within these channels in a manner analogous to layer intercalation in catalytic materials such as MoS_2_ [21]. An additional argument in support of channel bonding is the fact that the maximum concentrations of dye are less than those possible in palygorskite-based complexes [22] in which the dye is known to bond to the clay predominantly at surface sites.

## 5. Conclusions

In this paper we have presented a model for the chemical interaction between sepiolite and thioindigo in a clay/dye complex similar in nature to palygorskite/dye based Mayan pigment complexes. During synthesis sepiolite loses its zeolitic water content, opening up channels for the insertion of thioindigo molecules. The experimentally reported concentration of dye in this complex is consistent with channel absorption of the dye. Thioindigo attaches to the channel at an aluminum impurity site, with an additional but noninteracting aluminum impurity site nearby. The interaction is of a Van der Waals nature. The simulated model showed a significant distortion of the planar geometry of thioindigo, a characteristic of the well-known color changes in Mayan pigment-type materials. The UV/Vis spectrum of the model was simulated using both DFT and MO methods and is in excellent agreement with experimental results for the spectrum. Future studies will develop a model for a sepiolite/indigo complex.

## References

[1] Yacaman MJ, Puche MCS (1995). High resolution electron microscopy of maya blue paint. *Materials Research Society Symposium Proceedings*.

[2] Kleber R, Masschelein-Kleiner L, Thissen J (1967). Étude et identification du Bleu Maya. *Studies in Conservation*.

[3] Merwin HE (1931). *Temple Warriors at Chitzen Itza*.

[4] Gettens RJ, Stout GL (1946). *Paint Materials: A Short Encyclopedia*.

[5] Van Olphen H (1966). Maya Blue: a clay-organic pigment?. *Science*.

[6] Chianelli RR, Polette LA Color Compositions.

[7] Ramírez A, Sifuentes C, Manciu FS, Komarneni S, Pannell KH, Chianelli RR (2011). The effect of Si/Al ratio and moisture on an organic/inorganic hybrid material: thioindigo/montmorillonite. *Applied Clay Science*.

[8] Alvarez A (1984). *Palygorskite-Sepiolite: Ocurrences, Genesis, and Uses*.

[9] Bish DL, Guthrie GD Mineralogy of clay and zeolite dusts (exclusive of 1 : 1 layer silicates). *Reviews in Mineralogy*.

[10] Hansen K, Mossman BT (1987). Generation of superoxide (O2−_·_) from alveolar macrophages exposed to asbestiform and nonfibrous particles. *Cancer Research*.

[11] Ovarlez S, Giulieri F, Delamare F, Sbirrazzuoli N, Chaze AM (2011). Indigo-sepiolite nanohybrids: temperature-dependent synthesis of two complexes and comparison with indigo-palygorskite systems. *Microporous and Mesoporous Materials*.

[12] Giustetto R, Seenivasan K, Bordiga S (2010). Spectroscopic characterization of a sepiolitebased
Maya Blue pigment. *Periodico di Mineralogia*.

[13] Polette-Niewold LA, Manciu FS, Torres B, Alvarado M, Chianelli RR (2007). Organic/inorganic complex pigments: ancient colors maya blue. *Journal of Inorganic Biochemistry*.

[14] Ovarlez S, Chaze AM, Giulieri F, Delamare F (2006). Indigo chemisorption in sepiolite. Application to Maya blue formation. *Comptes Rendus Chimie*.

[15] Beaudoin JJ, Grattan-Bellew PE (1980). Collapse of structure in sepiolite and other layered silicate systems. *Cement and Concrete Research*.

[16] Payne MC, Teter MP, Allan DC, Arias TA, Joannopoulos JD (1992). Iterative minimization techniques for ab initio total-energy calculations: molecular dynamics and conjugate gradients. *Reviews of Modern Physics*.

[17] Stich I, Payne MC, King-Smith RD, Lin J-S, Clarke LJ (1992). Ab initio total-energy calculations for extremely large systems: application to the Takayanagi reconstruction of Si(111). *Physical Review Letters*.

[18] Hafner J (2008). *Ab-initio* simulations of materials using VASP: density-functional theory and beyond. *Journal of Computational Chemistry*.

[19] Zerner M (1991). *Reviews in Computational Chemistry*.

[20] Metropolis N, Rosenbluth AW, Rosenbluth MN, Teller AH, Teller E (1953). Equation of state calculations by fast computing machines. *The Journal of Chemical Physics*.

[21] Dungey KE, Curtis MD, Penner-Hahn JE (1998). Structural characterization and thermal stability of MoS2 intercalation compounds. *Chemistry of Materials*.

[22] Ovarlez S, Chaze AM, Giulieri F, Delamare F A chemical comprehension of the colour centres in Maya blue green palette. A study of indigo chemisorption in sepiolite.

